# Undergraduate Student Experience and Motivation Influence Perceived Benefits of Participation in Community Outreach

**DOI:** 10.59390/001c.146556

**Published:** 2025-12-31

**Authors:** Jason Martin, Davis Reina-Guerra, Leslie Stone-Roy

**Affiliations:** 1 Biomedical Sciences Colorado State University; 2 Anatomy & Neurobiology Boston University

**Keywords:** Brain Awareness Week, volunteer motivation, academic experience, teaching experience, education, high school, outreach

## Abstract

Community outreach benefits undergraduate students by providing opportunities to deepen content knowledge, practice teaching, develop a sense of scientific authority, and enhance communication skills. To evaluate whether students with varying academic levels or prior teaching experience differ in their motivations for volunteering and perceived benefits from Brain Awareness Week (BAW) outreach, we administered entry and exit surveys to undergraduate volunteers. The surveys included quantitative and qualitative questions to assess demographics, motivations, and prior outreach and teaching experience. Factor analysis of motivation items identified three constructs: career development, communication training, and STEM advocacy. Most students reported volunteering because they valued community outreach, enjoyed neuroscience, and/or wanted to enhance their resume / CV or applications. Lower-level students (freshmen and sophomores) were more motivated by career development (*p* = 0.014, *d* = 0.68) and reported greater need for communication training (*p* = 0.011, *d* = 0.69) compared to upper-level students. STEM advocacy scores were significantly higher among students with prior teaching experience, regardless of academic level (*p* = 0.001, *d* = 0.84). Exit surveys indicated that career development was positively correlated with increased confidence in approaching faculty (*r* = 0.58, *p* = 0.006) and communicating with diverse audiences (*r* = 0.56, *p* = 0.005). These findings align with established literature on the benefits of outreach and support tailoring recruitment and training strategies based on students’ prior experience to enhance both engagement and educational outcomes.

## Introduction

Supporting local communities by providing scientific outreach to K-12 schools is a common goal for many universities and colleges. Known benefits for elementary, middle and high schools include better understanding of scientific concepts and science relevance, increased interest in and enthusiasm for science, and increased interest in and enthusiasm for scientific career paths (Laursen et al., 2007). Another important benefit is increasing student interest in STEM (Crawford et al., 2021; Vennix et al., 2018). This can develop both as students are introduced to exciting and interesting aspects of science and when students see slightly older peers excited and knowledgeable about topics they may have thought were too difficult for them. Like high school students, university students report increased understanding and interest in specific topics after helping with outreach programs (Martins & Mello-Carpes, 2014). Additionally, university students learn the importance of giving back to the community, develop and practice skills such as teaching, and network with local teachers, peers, and university faculty (Deal et al., 2014; Hendrickson et al., 2020; Vollbrecht et al., 2019). University students also learn how to talk to the public and communicate concepts without relying on jargon or complicated terminology. The outreach experience of both the high school and university students helps produce educated adults that are able to convey the importance of science to lay people (Friedman, 2008). An additional documented benefit for university students is the contribution toward developing a science identity, which allows an individual to feel like an authority and therefore a true scientist (Graham et al., 2013). Thus, outreach programs like Colorado State University’s (CSU) Brain Awareness Week benefit both the K-12 population and the university students that provide the outreach experience in local schools (Laursen et al., 2007; Varner, 2014; Vollbrecht et al., 2019).

Brain Awareness Week (BAW) is a global program organized and supported by the Dana Foundation (https://www.brainawareness.org/ ). The goal of the program is to encourage interest in, and support for, neuroscience and research. The Dana Foundation and the Society for Neuroscience provide some resources and information about the program, and the Colorado State University BAW team organizes local neuroscience outreach events at middle and high schools. The Department of Biomedical Sciences at Colorado State University has held BAW events annually for over 15 years, except for 2020-2022 during the Covid-19 pandemic. Typically, over 100 CSU volunteers visit 3-5 middle and high schools during March and April. The success of the program can be inferred from the repeated requests to visit individual schools, and from the repeated participation of numerous CSU student volunteers. The program provides hands-on training for volunteers to support participation of undergraduate students at any stage of their higher education. The varied demographics among university student volunteers led to questions about whether academic experience and/or class standing impact motivation to participate in outreach, and/or the perceived outcomes of the outreach experience. Specifically, we predicted that students at higher levels (juniors and seniors) and those with prior teaching experience would volunteer for different reasons than lower-level students (freshman and sophomores) and those with no teaching experience. Likewise, we predicted that the perceived benefits of the experience would also differ between different groups.

To test these hypotheses, we designed entry and exit surveys to address the following:

1. Do students with different levels of academic, or teaching experience volunteer for different reasons?
2. Do the reported benefits of BAW volunteers differ based on academic level or prior teaching experience?
3. Are motivations to volunteer associated with perceived benefits of BAW participation? In addition to these questions, we gathered demographic data from our volunteers to assess reported identity characteristics.

## Methods

### Preparation for Events

The current study was based on 4 different events during one calendar year. The BAW director worked with teachers from 3 individual public schools to determine dates and times for each event. Four separate events were held, 3 during March-April, and 1 during November of the same year. Teachers were provided with a list and brief description of potential stations that were created previously by faculty and students at CSU. Each station covered a specific topic in neuroscience. The teachers and the director worked together to select 6-12 stations for each event. Stations covered a range of topics including, but not limited to: Human Brain Anatomy, Comparative Brain Anatomy, Chemical Senses, Visual Perception and Optical Illusions, Stroke, Epilepsy, and Neuroscience and Technology. Individual stations included 1-3 CSU student volunteers, a poster with information about the topic, and 1-3 hands-on activities. Funding for supplies and volunteer t-shirts was provided by the College of Veterinary Medicine and Biomedical Sciences, The Department of Biomedical Sciences, and The Molecular, Cellular, and Integrative Neurosciences Special Academic Unit at CSU. In addition, the Dana Foundation supplied some handouts including brain erasers and pencils.

### Surveys

The entry and exit surveys for volunteers were developed by the BAW director and included both quantitative and qualitative questions. The entry survey is presented in supplemental table 1 and the exit survey in supplemental table 2. At the beginning of each survey, CSU students were asked to create a code for identification so that entry and exit survey questions could be compared for individual student volunteers while maintaining anonymity. Surveys were approved by the CSU Institutional Review Board as exempt (protocol ID: 19-8713H). Incomplete surveys were excluded from analysis. In addition, if a volunteer participated more than once, only the initial entry and exit surveys were included in the study.

### CSU Student Volunteers (Volunteers)

Volunteers were recruited using online advertising through CSU, posted fliers and course announcements. Volunteers were required to attend an orientation session to learn about the program, expectations, and meet the program director. In addition, station titles and brief descriptions were provided so that volunteers could pick stations that most interested them. At the end of the orientation session, volunteers signed up to help with specific events, stations, date(s) and time(s). Subsequent training sessions were held for each station by the director, or by trained graduate students. Volunteers received a t-shirt that they were asked to wear at the event and then allowed to keep. Both entry and exit surveys were optional. Entry surveys were filled out either at the orientation session or at station-specific trainings. Exit surveys were filled out after individual events. Some students received course extra credit for volunteering, however it was not necessary to fill out the surveys to receive extra credit.

### Events

For this study, data were obtained from BAW activities that were held at two high schools and one middle school during a sample year. Events were two full days at one of the high schools and the middle school during March - April. A smaller event of one day with fewer classes was held at the second high school in April, and again in November of the same year. Thus, volunteers from 4 different BAW events were included in this study.

### Statistical Analyses

Statistical analyses of the entry and exit surveys were completed using Microsoft Excel, version 16.88 and IBM SPSS Statistics, version 29.0.2.0 for Apple macOS 15.0. Assumptions were met prior to performing all statistical analysis. Pearson and Spearman’s rho were calculated to identify correlations between variables. Both describe monotonic relationships in which values consistently increase (positive correlation) or decrease (negative correlation); strength and direction is reported on a scale from –1.00 to +1.00, with -1.00 representing a perfect negative correlation, 0.00 representing no association, and 1.00 representing a perfect positive correlation. Pearson *r* measures the strength and direction of a linear relationship between two parametric, normally distributed variables. In contrast, Spearman’s rho is a non-parametric measure used for ordinal data or for scale variables that are not normally distributed. To determine which correlation type was appropriate, variables were first evaluated using histograms to assess symmetry and if asymmetry was not visually apparent, then tested for skewness. Distributions with skewness values less than |1.00| were treated as parametric (*r*) and those greater than |1.00| were treated as non-parametric (*r_s_*). An initial exploratory analysis was conducted to examine associations between categorical demographic variables and the selection of individual motivation items (yes/abstain) using a Fisher’s exact test. This method was chosen because the data did not meet the minimum expected cell frequency assumption for a Pearson’s Chi-Square test, making the Fisher’s exact test more appropriate for the sample size; as this part of the analysis was exploratory, a decision was made not to apply a correction for multiple comparisons at this stage to reduce the risk of a Type II error (false negatives). Following this, a principal axis factor analysis with varimax rotation was used to group the motivation items into internally consistent constructs, which were confirmed with Cronbach’s Alpha. To compare the mean scores of these constructs between sub-populations, a series of independent t-tests was performed. To control the family-wise error rate and reduce the risk of a Type I error (false positives). for these comparisons, a Bonferroni correction was applied. The analysis involved two separate “families” of tests (one for each sub-population). Because three constructs were tested within each family, the alpha level for statistical significance was adjusted to *p* < .017 (.05/3). A result described as “more likely than chance” signifies a rejection of the null hypothesis, which assumes no association between variables. (Cohen, 1988) and (Morgan et al., 2020) were used to interpret the effect sizes of each analysis.

## Results

Overall, over 100 CSU volunteers participated in BAW outreach during the time studied. As expected, not all volunteers filled out the optional surveys and different numbers filled out the entry and exit surveys. Entry surveys were filled out by 63 undergraduate students and exit surveys were filled out by 23 undergraduate students. Some students only filled out the entry or the exit survey, and 12 CSU student volunteers filled out both surveys. The data from 2 volunteers were eliminated because they didn’t fit into the available class rankings (freshman, sophomore, junior, senior, e.g., students earning a 2^nd^ bachelor’s degree).

## Volunteer demographics

Of the 61 surveys filled out by qualified undergraduate volunteers, 43.6% (*n* = 26/61) were classified as lower-level (freshman and sophomores / less than 60 semester credits completed) and 57.4% (*n* = 35/61) as upper-level (juniors and seniors / more than 60 semester credits completed). As stated previously, different numbers of students filled out the entry and exit surveys. The entry survey was completed by 97% (*N* = 59/61) of volunteers. Only the 59 volunteers who completed the entry survey provided the following demographic data. Two more surveys were eliminated at this point because they were incomplete. Most of the 59 volunteers identified as white (91.5%, *n* = 54/59) and more volunteers identified as female (55.9%, *n* = 33/59) than male (42.4%, *n* = 25/59). Volunteers reported a variety of undergraduate majors and career plans, with Biomedical Science (49.2%, *n* = 29/59) or Neuroscience (27.1%, *n* = 16/59) being the most represented majors and Medical School (39.0%, *n* = 23/59) and Unsure / Other (32.2%, *n* = 19/59) being the most reported career plans following graduation. While most volunteers had not participated in a prior BAW event (48/59, 81.4%), nearly half (n = 28/59, 47.5%) had prior STEM teaching experiences (e.g., tutoring other students, serving as a teaching assistant, or participating in other outreach events). Participant demographics by entry and exit survey are presented in Table 1.

**Table 1.** Self-Reported Volunteer Demographics.

		Entry Survey		Exit Survey	
	Response	*n^1^*	%	*n^2^*	%
Gender					
	Female	33	55.9%	10	40.0%
	Male	25	42.4%	12	48.0%
	Non-Binary or N/A	1	1.7%	3	12%
Race					
	White	54	91.5%	21	84.0%
	Asian	2	3.4%	1	4.0%
	Other or N/A	3	5.1%	3	12%
Ethnicity					
	Not Hispanic	51	86.4%	22	88.0%
	Hispanic	6	10.2%	0	0%
	N/A	2	3.4%	3	12%
Undergraduate Status					
	Lower-level	26	44.1%	7	28.0%
	Upper-level	33	55.9%	18	72.0%
Major					
	Biomedical Science	31	52.5%	12	48.0%
	Neuroscience	17	28.8%	6	24.0%
	Biology	4	6.8%	2	8.0%
					
	Other or N/A	7	11.9%	5	20.0%
Plans After Graduation					
	Medical School	23	39.0%	8	32.0%
	Graduate School	8	13.6%	5	20.0%
	Veterinary School	5	8.5%	1	4.0%
	Physician Assistant School	3	5.1%	3	12.0%
	Job	1	1.7%	0	0.0%
	Unsure, Other, or N/A	19	32.2%	8	32.0%
Previously Volunteered for Brain Awareness Week					
	Yes	11	18.6%	7	28.0%
	No	48	81.4%	16	64.0%
	N/A	0	0.0%	2	8.0%
Previously Taught Science					
	Yes	28	47.5%	14	60.9%
	No	28	47.5%	8	39.1%
	N/A	3	5.1%	3	4.2%

*N^1^* = 59, *N^2^* = 25

Lower-level: < 60 semester credits completed

Upper-level: ≥ 60 semester credits completed

N/A: No Answer

### Measuring Motivation to Volunteer

To help identify reasons why CSU students volunteer to help with BAW outreach, the entry survey included a list of 11 motivation items and volunteers were asked to choose the items that applied to them (Supplemental table 1, question 9). All but one of the options (as part of an honor’s thesis) were selected by some students. Notably, three of the options were selected by at least 70% volunteers. These were: 1) *because I think outreach/community service is important* (*n* = 47/59, 79.7%), 2) *because I enjoy neuroscience* (*n* = 45/59, 76.3%), and 3) *to add outreach to my resume, CV, or future applications* (*n* = 42/59, 71.2%). The other 7 motivation items were selected by 3-59% of the volunteers, depending on the specific statement (Table 2).

**Table 2.** Motivation Index Counts and Percentages for Volunteering for Brain Awareness Week

Why did you volunteer for BAW? Check all that apply.	Indicated	%
Because I think outreach/community service is important	47	79.66
Because I enjoy neuroscience	45	76.27
To add outreach to my resume, CV or future applications	42	71.19
To learn more about neuroscience	35	59.32
To work more closely with faculty	29	49.15
To see if I can teach &/or if I enjoy teaching	23	38.98
To work more closely with other CSU students	23	38.98
To earn extra credit for a class	21	35.59
I used to go to the middle or high school and wanted to give back or visit	4	6.78
Other.	2	3.39
As part of an honor’s thesis or other thesis (I created a new station)	0	0.00

*N* = 59

BAW: Brain Awareness Week

CV: Curriculum Vitae

CSU: Colorado State University

To investigate whether differences in motivation item counts (reported as either a response indicating yes or abstaining implying no) could be explained by demographics (specific sub-population of participants compared with all other participants), a Fisher’s exact test was performed for each item. Volunteers with science teaching experience were more likely than chance to report that they were motivated to participate *to work more closely with other CSU students* (p = 0.015). In contrast, for undergraduate class, gender, race, and ethnicity, the threshold for statistical significance was not reached (*p* > .05) and it was determined that for those variables no motivational item was more likely than chance to be selected.

Select courses offered extra credit for outreach events like BAW and 21/59 (35.6%) of volunteers received extra credit for participation in the program. Most of these, (19/21, 90.5%) were biomedical science majors, with the remaining two volunteers reporting neuroscience and biology majors. The entry survey asked volunteers if they expected to receive extra credit for their participation in BAW, and of the 21 that said yes, less than a quarter of the volunteers (5/21, 23.8%) reported that extra credit influenced their decision to participate. Neither those with experience teaching science (*p* = .697) nor upper-level volunteers (*p* = .466) were more likely than chance to be motivated by extra credit.

To further investigate the reasons why students volunteer for outreach, the entry survey included a list of items associated with a Likert-scale ranging from *strongly disagree* (1) to *strongly agree* (6). In addition, “not applicable” was included as option 7 (supplemental table 1, question 12). To determine if specific questions could be grouped, a principal axis factor analysis (varimax rotation) was performed on 11 six-point Likert scale items included in the entry survey. This analysis resulted in the identification of 3 groups, which accounted for 52.3% of the variance (Table 3). Based on the grouped questions, 3 constructs were identified and labeled: 1. *career development* (20.3% of the variance), 2. *communication training* (17.1%), and 3. *STEM advocacy* (14.9%). Questionnaire items and rotated factor loadings greater than |.40| are presented in Table 3. One item (*I feel confident teaching science to younger students*) was associated with both factors 2 and 3 and was assigned to factor 3 because it had the larger absolute value indicating a stronger relationship. Constructs were formed by averaging the composite variables associated with item responses. Cronbach’s Alphas of .79, .79, and .67 were calculated for factors 1, 2, and 3, respectively, indicting strong internal consistency for each factor. The identification of constructs allowed more efficient comparison between upper and lower-level students, and those with, or without, prior teaching experience. For each of the three constructs, a construct score was calculated by taking the average prompt response for included questions.

**Table 3.** Rotated Factor Loadings from a Principal Factor Analysis with Varimax Rotation of the Entry Survey’s “Motivation Questions” scale with Cronbach’s Alphas

Item	Factor Loadings		
	1	2	3
I hope volunteering for BAW will help define my career path.	0.89		
I hope volunteering for BAW will expand my academic network.	0.72		
I hope volunteering will help me talk about science to others.	0.67		
I expect to learn more about neuroscience by volunteering for BAW.	0.53		
I need training or education about how to talk to MS and HS students.		0.84	
I need training or education about how to teach.		0.69	
I need training about interacting with students different from me.		0.64	
I learn better if I can apply knowledge.			0.64
I feel confident teaching science to younger students.		-0.42	0.63
I like teaching.			0.61
I think community outreach is important.			0.52
			
% of variance	20.32	17.13	14.87
			
Cronbach's Alpha	.79	.79	.67

1: Career Development

2: Communication Training

3: STEM Advocacy

*N* = 59; Omitted: loadings < |0.40|

### Measuring perceived benefits after the volunteer experience

After completing the BAW event, student volunteers were asked to complete an exit survey (supplemental table 2) which included nine 6-point Likert-scale outcome-focused prompts, ranging from *strongly disagree* (1) to *strongly agree* (6). Twenty-five of the volunteers completed the exit survey. The data from 2 volunteers were eliminated because they did not complete an entry survey in addition to the exit survey. To better understand volunteer responses, the three construct scores calculated from the entry survey were correlated with exit survey prompt responses (Table 4). The Career Development construct was positively correlated with the questions “*volunteering for BAW helped me feel more comfortable approaching faculty members at CSU*” (Pearson correlation, *r* = .58, *p* = .006) and “*I feel more confident in my ability to communicate with different types of people after helping with BAW*” (*r* = .56, *p* = .005). In contrast, the Career Development construct was negatively correlated with the prompt “*while volunteering I looked at other stations and / or talked to other volunteers*.” A negative correlation was calculated for Communication Training and the prompts “*I learned something new about neuroscience through the BAW experience*” (Spearman’s rho, *r_s_* = -.52, *p* = .014), “*I thought about how to present my station before volunteering*” (*r_s_* = -.48, *p* = .025), and “*I enjoyed being a role model for Middle School and High School students*” (*r_s_* = -.43, *p* = .041). Only one exit survey prompt, “I am more interested in incorporating teaching into my career plans after volunteering” (*r* = .52, *p* = .010), was correlated with the STEM Advocacy construct created from the entry survey data. Exit-survey questions were considered normally distributed if their skewness was < |1| and no anomalies were present on a histogram.

**Table 4.** Exit survey responses showed correlations with the Career Development, Communication Training and STEM Advocacy constructs developed from entry survey responses.

			Career Development (4 Items)		Communication Training (3 Items)		STEM Advocacy (4 Items)	
	*n*	*Skewness*	*r / r_s_*	*p*	*r / r_s_*	*p*	*r / r_s_*	*p*
Volunteering for BAW helped me feel more comfortable approaching faculty members at CSU	21	0.06	**0.58**	**0.006**	-0.18	0.439	0.35	0.116
I feel more confident in my ability to communicate with different types of people after helping with BAW	23	-0.85	**0.56**	**0.005**	0.10	0.640	0.08	0.715
While volunteering I looked at other stations and / or talked to other volunteers	22	-1.08	**-0.53**	**0.011**	0.06	0.796	-0.25	0.261
I learned something new about neuroscience through the BAW experience	22	-0.33	0.05	0.830	**-0.52**	**0.014**	0.12	0.582
I thought about how to present my station before volunteering	22	-1.23	-0.15	0.504	**-0.48**	**0.025**	0.39	0.074
I enjoyed being a role model for MS and HS students	23	-1.30	-0.15	0.491	**-0.43**	**0.041**	-0.06	0.782
I am more interested in incorporating teaching into my career plans after	23	-0.51	0.11	0.610	-0.10	0.642	**0.52**	**0.010**
During BAW volunteering, I realized that I know more than I thought about neuroscience	23	-1.46	0.38	0.072	-0.13	0.562	0.39	0.065
I feel like I had an impact on at least one student while volunteering	23	-1.86	0.03	0.884	-0.18	0.412	0.21	0.328

*r = Pearson correlation

r_s_*= *Spearman’s rho*

Differences in career development and communication training constructs were noted based on undergraduate status, and a difference in STEM advocacy was associated with science teaching experience (Table 5). Lower-level volunteers were found to have greater career development (*t*(57) = 2.54, *p* = .014, *d* = .68) and communication training (*t*(57) = 2.64, *p* = .011, *d* = .69) constructs than upper-level participants, but the groups were not statistically different with respect to STEM advocacy (*t*(57) = -1.21, *p* = .231, *d* = .32. In contrast, volunteers with prior science teaching experience had greater STEM advocacy constructs (*t*(54) = 3.155, *p* = .001, *d* = .84) than those who didn’t, but no statistically significant difference in career development (*t*(54) = -1.24, *p* = .219, *d* = .07) or communication training (*t*(54) = -.26, *p* = .793, *d* = .33). The effect size *d* is considered typical if greater than |.5| and large if greater than |.8| (Cohen, 1988; Morgan et al., 2020).

**Table 5.** Comparison of Volunteer Career Development, Communication Training, and STEM Advocacy Constructs by Undergraduate Student Level and Prior Science Teaching Experience

	Variable		*M*	*SD*	*t*	*df*	***α***	*d*
Undergraduate Status	Career Development				2.54	57	0.014	0.68
		Lower-level	5.45	0.58				
		Upper-level	5.00	0.73				
	Communication Training				2.64	57	0.011	0.69
		Lower-level	3.38	1.16				
		Upper-level	2.59	1.15				
	STEM Advocacy				-1.21	57	0.231	0.32
		Lower-level	5.42	0.44				
		Upper-level	5.55	0.40				
Science Teaching Experience	Career Development				-0.26	54	0.793	0.33
		Prior Volunteer	5.18	0.70				
		Novice Volunteer	5.23	0.74				
	Communication Training				-1.24	54	0.219	0.07
		Prior Volunteer	2.75	1.06				
		Novice Volunteer	3.15	1.36				
	STEM Advocacy				3.155	54	0.001	0.84
		Prior Volunteer	5.66	0.32				
		Novice Volunteer	5.34	0.44				

*M* = Mean, *SD* = Standard Deviation, *df* = Degrees of Freedom **α = Bonferroni correction (significance at α < .017)**

## Discussion

University students benefit from participating in community outreach, which increases interest in science, develops communication skills, provides practice teaching, as well as exposes students to public perceptions of science (Martins & Mello-Carpes, 2014; Saravanapandian et al., 2019; Vollbrecht et al., 2019). In addition, these experiences increase student science knowledge and confidence, thus enhancing the development of their science identity, which is defined as the feeling of being an authority in science and belonging to the science community (Gubbels & Vitiello, 2018; Stevens, 2011). Just as important, these experiences produce adults with science knowledge who will integrate into many different jobs, contributing to a more educated public (Stevens, 2011). Although the benefits of engaging in outreach have been studied, there is less information about why undergraduate students participate in outreach and whether student experience affects participation or perceived benefits. This study aimed to determine the motivations and perceived benefits for undergraduate students volunteering in Brain Awareness Week outreach. It also tested whether prior experience, as defined by class standing (lower-level = freshman and sophomores or higher-level = juniors and seniors) and/or prior STEM teaching experience (e.g., as a teaching assistant, or a tutor), were/was associated with reasons for volunteering or perceived benefits. Our results indicate that university students participate in BAW outreach for various reasons. Over 70% of volunteers reported that they volunteered because they consider community outreach important, they enjoy neuroscience, and/or they wanted to add outreach to their resume, CV, or future applications. Forty-nine to fifty nine percent volunteered to work more closely with faculty and learn more about neuroscience. These results are consistent with the limited literature investigating student motivations for outreach participation. For example, previous studies demonstrated that undergraduate students are strongly motivated to participate in community outreach partly due to a belief that community outreach is important for the students themselves, the community, and the university. The enjoyment of neuroscience and the opportunity to increase scientific knowledge as motivators have also been reported (Vollbrecht et al., 2019). With respect to student volunteers reporting that they wanted to enhance applications and resumes, our recruiting verbiage included this potential benefit, so the strong response agreeing with this statement is not surprising.

To determine if students with varied academic experiences have different motivations to participate in outreach, we tested the hypothesis that more experienced students would volunteer for different reasons than those with less experience. To explore this, we analyzed Likert-scale questions that resulted in the identification of 3 constructs: Career Development, Communication Training, and STEM Advocacy. These constructs were then used to determine whether prior experience was associated with reasons for volunteering. Our results indicated that lower-level students with less university experience (freshman and sophomores) were more likely to volunteer to enhance their career development and communication skills when compared to students with more university experience (juniors and seniors). There was no difference between lower and upper-level students with respect to STEM Advocacy. Analysis of students with different teaching experiences revealed that those with prior teaching experience were more likely to volunteer because they like or feel confident about teaching, feel that hands-on learning is effective, and/or think community outreach is important (STEM Advocacy Construct). In contrast, prior teaching experience didn’t affect the motivation to enhance career development or communication skills. Thus, our results were mixed. Experienced students had some differences with respect to why they volunteered for outreach, but in other cases their reasons were similar to less experienced students.

We next tested the hypothesis that the perceived benefits of the volunteering experience would differ based on class standing and/or prior teaching experience. Using the identified constructs from the pre-survey motivational analysis, we analyzed results from exit surveys after students had experienced volunteering. Undergraduate students at the beginning of their college career (Freshman and Sophomores), had greater career development and communication constructs relative to Juniors and Seniors. Lower-level students were more likely to report that the experience helped them feel more comfortable approaching faculty and communicating with different types of people. Multiple studies have shown that communication skills increase after undergraduates participate in outreach (Vollbrecht et al., 2019). Our study indicates that this benefit is especially important for students at the beginning of their college journey. Prior experience teaching is another form of academic experience, and we found that students with prior teaching experience had more STEM advocacy constructs compared to those with no experience, suggesting that prior experiences were associated with the increased perception that community outreach is important.

Undergraduate participation in community outreach contributes to the development of a science identity, and to retention of students in STEM Fields. Developing a science identity is an important part of the undergraduate experience for STEM students, and it refers to the ability of a person to feel like an expert or authority on a science topic, and a feeling of belonging to a science community (Huffmyer et al., 2022). Because CSU’s BAW program includes volunteers at all stages of their undergraduate careers, our students vary widely with respect to their progress toward developing a science identity. Volunteers who enjoy neuroscience have likely begun to form a science identity and are therefore more likely to feel confident enough to volunteer for the program (Gubbels & Vitiello, 2018; Murphy & Kelp, 2023). Because the act of participating in science outreach can enhance the formation of a student’s science identity, it is important to engage students with strong identities, and those who have not yet formed a strong science identity (Millar et al., 2019). Being motivated by the desire to add outreach to future application materials is also related to science identity since it indicates to others that a person has experience as a science authority. Similarly, working with science faculty helps students feel like a part of the science community. Thus, community outreach programs contribute to multiple aspects of an undergraduate college experience and retention of these programs is important (Friedman, 2008). Our research suggests that recruitment of undergraduate students into these programs should be tailored based on their level of experience as a university student, and as a teacher.

## Supplementary Material

Supplementary Materials 1

Supplementary Materials 2
