# Supplementary material for: Undergraduate Student Experience and Motivation Influence Perceived Benefits of Participation in Community Outreach
Source: J Undergrad Neurosci Educ. 2025 Dec 31;24(1):56–65. doi: 10.59390/001c.146556 (PMC13127667; doi:10.59390/001c.146556)
Supplement: Supplementary Materials 1 [file junejournal_2025_24_1_146556_308466.pdf]

Brain Awareness Week
Pre- Survey to help assess impact of experience on CSU student volunteers
Stone-Roy

Please fill out and answer the questions

1. Identification Code: _____________________________________. Please use the following code: the first 2 letters of your first name, the first 2 of your last name, month and day of birth. (e.g. Lest0205) This is so we can compare pre- and post- survey responses while still maintaining confidentiality.

2. CSU status (check one):
____ Undergraduate student. Circle one: freshman/sophomore/junior/senior
____ Graduate student. Degree working on: Masters ____ PhD ____ Other ____
____ Postdoctoral fellow
____ Staff
____ Community volunteer
____ Other (please describe) ____

3. Department, program or major at CSU _______________________________________

4. Gender:
____ Non-binary
____ Female
____ Male

5. Race:
____ White
____ Black or African American
____ American Indian or Alaska Native
____ Asian
____ Native Hawaiian or other Pacific Islander
____ Other Please describe:

6. Ethnicity:
____ Hispanic or Latino
____ Not Hispanic or Latino

7. What are your plans after graduation (e.g., job, graduate school, medical school, dental school, postdoctoral fellowship, faculty position at university, etc.)

8. What station & age group (middle or high school) are you volunteering to help with?

Why did you pick this station?

9. Why did you volunteer for BAW? Check all that apply.

____ To add outreach to my resume, CV or future applications
____ To earn extra credit for a class
____ To see if I can teach &/or if I enjoy teaching
____ To learn more about neuroscience
____ To work more closely with faculty
____ To work more closely with other CSU students
____ Because I think outreach/community service is important
____ Because I enjoy neuroscience
____ As part of an honor's thesis or other thesis (I created a new station)
____ I used to go to the middle or high school and wanted to give back or visit
____ Other. Please explain:

10. Have you volunteered for BAW before? How many times?

If you've volunteered for BAW before, please answer the following questions:

A. Why do you continue to volunteer (i.e., what do you get out of it that is important enough to spend time doing BAW outreach)

B. Do you volunteer for the same station each time, or different stations? Why?

11. Have you helped teach science before (e.g., experience as a teaching assistant, K-12 outreach that involved teaching, participation in Heartbreakers or Brainstorm at the museum, tutoring, etc.)

____ No ____ Yes. Please describe this experience:

12. Please respond to the statements below using the following scale:
strongly disagree = 1
moderately disagree = 2
slightly disagree = 3
slightly agree = 4
moderately agree = 5
strongly agree = 6
not applicable = 7

	Strongly disagree	moderately disagree	slightly disagree	slightly agree	moderately agree	Strongly agree	Not Applicable
	1	2	3	4	5	6	7
I expect to like volunteering for BAW	○	○	○	○	○	○	○
I find neuroscience difficult	○	○	○	○	○	○	○
I like neuroscience	○	○	○	○	○	○	○
I would like to learn more about neuroscience	○	○	○	○	○	○	○
I I find neuroscience interesting	○	○	○	○	○	○	○
I am a strong student academically	○	○	○	○	○	○	○
The grades I receive in lecture courses reflect my knowledge and abilities	○	○	○	○	○	○	○
I like teaching	○	○	○	○	○	○	○
I learn better in situations where I can apply knowledge	○	○	○	○	○	○	○
I think community outreach is important	○	○	○	○	○	○	○
I feel confident teaching science to younger students	○	○	○	○	○	○	○
I am the first person in my family to go to college							
My GPA is above 3.0	○	○	○	○	○	○	○

	Strongly disagree	moderately disagree	slightly disagree	slightly agree	moderately agree	Strongly agree	Not Applicable
	1	2	3	4	5	6	7
I will attend station specific training prior to volunteering	○	○	○	○	○	○	○
I will think about how to present my station prior to volunteering	○	○	○	○	○	○	○
I need training or education about how to teach	○	○	○	○	○	○	○
I like the idea of teaching middle or high school students in small groups rather than in a lecture setting	○	○	○	○	○	○	○
I understand that I will be representing CSU while volunteering	○	○	○	○	○	○	○
I need training or education about how to talk to middle or high schoolers	○	○	○	○	○	○	○
I need training or education about how to interact with students who are different from me (race, age, socioeconomic status, sexual orientation, etc)	○	○	○	○	○	○	○
I expect to better understand how to communicate with different types of people after BAW	○	○	○	○	○	○	○
I expect to learn more about neuroscience by volunteering for BAW	○	○	○	○	○	○	○
I hope volunteering for BAW will help me define my career path	○	○	○	○	○	○	○
I hope that volunteering will expand my academic network	○	○	○	○	○	○	○

	Strongly disagree	moderately disagree	slightly disagree	slightly agree	moderately agree	Strongly agree	Not Applicable
	1	2	3	4	5	6	7
I am volunteering for BAW only because I will get extra credit in a class	○	○	○	○	○	○	○
I hope volunteering will enhance my ability to talk about science with other people	○	○	○	○	○	○	○
I like the idea of being a role model for middle and high school students	○	○	○	○	○	○	○
I believe people learn more about topic if they teach it to another person instead of just learning it for themselves	○	○	○	○	○	○	○

Is there anything else you hope to get out of volunteering for Brain Awareness Week activities?

If yes, please explain:

Additional Comments:
