# Supplementary material for: Undergraduate Student Experience and Motivation Influence Perceived Benefits of Participation in Community Outreach
Source: J Undergrad Neurosci Educ. 2025 Dec 31;24(1):56–65. doi: 10.59390/001c.146556 (PMC13127667; doi:10.59390/001c.146556)
Supplement: Supplementary Materials 2 [file junejournal_2025_24_1_146556_308467.pdf]

Brain Awareness Week
Post-volunteering Survey to help assess impact of experience on CSU student volunteers
Stone-Roy

Please fill out and answer the questions

1. Identification Code: ______________________________________. Please use the same code you did for the pre-survey: the first 2 letters of your first name, the first 2 of your last name, month and day of birth. (e.g. Lest0205) This is so we can compare pre- and post-survey responses while still maintaining confidentiality.

2. What station & age group (middle or high school) did you volunteer to help with?

3. Please select your role in BAW circle all that apply: Volunteer Station manager

4. If you managed a station, was there anything valuable about that experience?

5. Did your volunteer experience change any ideas you have about future plans? Please explain.

6. What aspects of volunteering for BAW did you enjoy or think were valuable?

7. What aspects of volunteering for BAW need to be improved or did you find frustrating or annoying?

8. If you've volunteered for BAW before this year, please answer the following questions (I'm asking again in this survey in case your more recent experience reminded you of anything you forgot to mention on the pre-survey):

A. Why do you continue to volunteer for BAW (i.e., what do you get out of it that is important enough to spend time)

B. Did you volunteer for the same station as before, or a different station? If different, please list stations

9. In your opinion, what do the middle or high school students get out of the BAW experience?

10. Please respond to the statements below using the following scale:

strongly disagree = 1
moderately disagree = 2
slightly disagree = 3
slightly agree = 4
moderately agree = 5
strongly agree = 6
not applicable = 7

	Strongly disagree	moderately disagree	slightly disagree	slightly agree	moderately agree	Strongly agree	Not Applicable
	1	2	3	4	5	6	7
I enjoyed volunteering for BAW	○	○	○	○	○	○	○
I learned something new about neuroscience through the BAW experience	○	○	○	○	○	○	○

	Strongly disagree	moderately disagree	slightly disagree	slightly agree	moderately agree	Strongly agree	Not Applicable
	1	2	3	4	5	6	7
I learned something new about teaching others while volunteering at BAW	○	○	○	○	○	○	○
I became interested in a new or different neuroscience topic because of my volunteering	○	○	○	○	○	○	○
I am more interested in incorporating teaching into my career plans than I was prior to BAW	○	○	○	○	○	○	○
The students at the middle or high school seemed to enjoy the BAW station I helped with	○	○	○	○	○	○	○
The students at the middle or high school seemed to be interested in what I was teaching them	○	○	○	○	○	○	○
The students at the middle or high school seemed to like the station activity	○	○	○	○	○	○	○
I enjoyed teaching the middle or high school students	○	○	○	○	○	○	○
I feel volunteering for BAW was a valuable experience	○	○	○	○	○	○	○
I feel like I had an impact on at least one student while volunteering	○	○	○	○	○	○	○
I felt confident teaching science to the younger students	○	○	○	○	○	○	○
I invited friends to volunteer for BAW	○	○	○	○	○	○	○

	Strongly disagree	moderately disagree	slightly disagree	slightly agree	moderately agree	Strongly agree	Not Applicable
	1	2	3	4	5	6	7
I attended station specific training prior to volunteering	○	○	○	○	○	○	○
The person that trained me for my station did a good job	○	○	○	○	○	○	○
I thought about how to present my station prior to volunteering	○	○	○	○	○	○	○
The person who trained me covered the scientific aspects of the station and activity well	○	○	○	○	○	○	○
The person who trained me explained how to talk to and engage middle or high school students	○	○	○	○	○	○	○
While at the middle or high school, I looked at other stations or talked to other volunteers during breaks	○	○	○	○	○	○	○
I talked to the teachers at the middle or high school	○	○	○	○	○	○	○
I was comfortable interacting with students who were different from me (race, age, socioeconomic status, sexual orientation, etc)	○	○	○	○	○	○	○
I feel more confident in my ability to communicate with different types of people after helping with BAW	○	○	○	○	○	○	○
Volunteering for BAW helped me feel more comfortable approaching faculty members at CSU	○	○	○	○	○	○	○
CSU should continue to support Brain Awareness Week activities	○	○	○	○	○	○	○

	Strongly disagree	moderately disagree	slightly disagree	slightly agree	moderately agree	Strongly agree	Not Applicable
	1	2	3	4	5	6	7
I will receive extra credit in a course for participating in BAW	○	○	○	○	○	○	○
I feel that teaching neuroscience to other students helped solidify some concepts for me or helped me understand specific concepts better	○	○	○	○	○	○	○
I enjoyed being a role model for middle and high school students	○	○	○	○	○	○	○
During BAW volunteering, I realized that I know more than I thought about neuroscience	○	○	○	○	○	○	○

Is there anything else you got out of volunteering for Brain Awareness Week activities?

Do you have any suggestions for specific stations or the overall program?

Additional Comments:
